# Incidence, characteristics and risk factors of thromboembolic events in East Asian patients with *BCR-ABL1* negative myeloproliferative neoplasms

**DOI:** 10.1038/s41598-021-97464-4

**Published:** 2021-09-08

**Authors:** Jinyong Kim, Ja Min Byun, Junshik Hong, Youngil Koh, Dong-Yeop Shin, Inho Kim, Sung-Soo Yoon, Hyunkyung Park, Soo-Mee Bang

**Affiliations:** 1 Department of Internal Medicine, Seoul National University College of Medicine, Seoul National University Hospital, 101 Daehak-ro, Jongno-gu, Seoul, 03080 Korea; 2 Cancer Research Institute, Seoul National University College of Medicine, Seoul, 110744 Korea; 3 Center for Medical Innovation, Biomedical Research Institute, Seoul National University Hospital, Seoul, 110744 Korea; 4 Department of Internal Medicine, Seoul National University Boramae Medical Center, Seoul, 07061 Korea; 5 Department of Internal Medicine, Seoul Nationl University College of Medicine, Seoul National University Bundang Hospital, Seongnam, 463-707 Korea

**Keywords:** Myeloproliferative disease, Cancer epidemiology

## Abstract

The vascular complications have been a major cause of morbidity and mortality among all subtypes of *BCR-ABL1* negative myeloproliferative neoplasms (MPN), but the ethnicity-specific data was limited. We therefore conducted a multi-center retrospective, longitudinal cohort study to evaluate the incidence, characteristics and risk factors of thromboembolic events of MPN patients. Of 256 patients, 27.3% experienced thromboembolic events, majority of which occurred before or within 12 months of MPN diagnosis. The multivariable Cox proportional analyses identified leukocytosis (HR 2.67, 95% CI 1.36–5.24, q = 0.004) and history of thrombosis (HR 9.68, 95% CI 2.00–46.88, q = 0.005) as the risk factors for thromboembolism. In subgroup analysis of polycythemia vera and hemoglobin concentration (HR 1.97, 95% CI 1.28–3.04, q = 0.002) appeared to be a significant risk factor of thrombosis, along with age and thrombosis history. In essential thrombocythemia, severity of the established IPSET score was closely correlated with the frequency of thromboembolic events. In primary myelofibrosis, history of thrombosis was associated with thrombosis events (HR 13.85, 95% CI 1.2–159.5, q = 0.035). Overall survival was worse in patients who experienced thromboembolic events. Our study highlighted the importance of recognizing high risk patients and implementing personalized intervention.

## Introduction

*BCR-ABL1* negative myeloproliferative neoplasms (MPN) represent a heterogeneous group of clonal hematopoietic cells, comprising polycythemia vera (PV), essential thrombocythemia (ET), and primary myelofibrosis (PMF)^1^. Despite the relatively indolent clinical course, many patients with PV or ET experience arterial or venous thrombosis attributed to high blood viscosity^2^. In patients with PMF, excessive inflammatory cytokines release results in fibrotic changes in bone marrow, vascular complications and constitutional symptoms^3^. All in all, thromboembolic events are a major cause of morbidity and mortality among all subtypes of MPN^4,5^.

The discovery of the *JAK2V617F* mutation has allowed for development of novel therapeutic agents and has encouraged the efforts towards molecular diagnostics for MPN^6–8^. Despite the better understanding of the disease however, data regarding the disease course of MPN in Asian populations remain scarce. Recently three South Korean epidemiological studies showed that the incidence and prevalence of MPN is on the rise at a rate of 3.8 times in last ten years^9–11^. With such increment, MPN impose a cumulative threat to public health by inducing substantial economic and social burdens. Given that, understanding the incidence and risk factors for major complications of MPN, namely thromboembolic events, is important. Previous studies have reported the prevalence of thrombosis among MPN patients was 20% to 30% or more, which can occur throughout the disease course ^12,13^. More specifically, a recent meta-analysis of 29 cohort studies including populations of Europe, North America, Asia, and Australia reported that the pooled prevalence of arterial or venous thrombosis among MPN patients at diagnosis was 20% (95% CI, 16.6–23.8%)^12^. In German study, 33.6% suffered from vascular events throughout the disease course^13^. Asian population has traditionally been associated with lower incidence of idiopathic thromboembolism and certain types of cancer-associated thromboembolisms compared to other ethnicities^14–16^. Recognizing the paucity of data on thromboembolic events in Asian MPN patients, we conducted this study to find out the incidence, characteristics, and risk factors of thromboembolic events in Asian MPN patients.

## Materials and methods

### Study design and subjects

This was a multi-center retrospective, longitudinal cohort study of *BCR-ABL1* negative MPN patients over 18 years old. The study period was set between January 2008 and December 2018. From 2008 to 2015, the diagnosis of MPN was made according to the 2008 World Health Organization (WHO) classification. From 2016 to 2018, the diagnosis of MPN was made according to the revised 2016 WHO classification. At first 406 patients were identified, but after excluding patients without bone marrow examination results (n = 22), those without JAK2 V617F mutational status and/or blood counts at diagnosis (n = 100), and those diagnosed before 2008 (n = 28), 256 patients were finally included for analysis. Other driver mutational status, such as *JAK2* exon 12, *CALR* and *MPL*, was checked if clinically indicated. Their medical records were reviewed and analyzed for demographics, disease characteristics, treatment including cytoreductive, antiplatelet and anticoagulation therapy, and clinical course. The study was conducted according to Declaration of Helsinki and was approved by the institutional review board (IRB) of each hospital (Seoul National University Hospital IRB, IRB approval number J-1809-006-968; Seoul National University Bundang Hospital IRB, IRB approval number B-1809-492-404; Seoul National University Boramae Medical Center IRB, IRB approval number 20-2018-50). All patient data were anonymized and de-identified prior to analysis, and thus the requirement for patient consent was waived by the IRB of all hospitals (same as above).

### Definitions

Cardiovascular disease (CVD) risk factors were obesity with BMI over 25, smoking, hypertension, diabetes, and dyslipidemia. Thrombosis was categorized into arterial or venous thromboembolisms. Arterial thrombosis included acute coronary syndrome (ACS), stroke and peripheral artery disease (PAD). Venous thromboembolism included deep vein thrombosis (DVT), pulmonary embolism (PE) and splanchnic vein thrombosis (SVT). Other minor occlusive events, including stable angina and superficial thrombophlebitis were not included.

The variables clinically relevant to thrombosis were defined per NCCN Guidelines®^17^. Clinically relevant risk factors included age at diagnosis, sex, previous thrombotic event, hepatomegaly, splenomegaly, WBC > 15 × 10^9^/L, hemoglobin count, platelet count, *JAK2 V617F* mutation status, and two or more of the cardiovascular risk factors mentioned above.

The presence of thromboembolic event was confirmed with an imaging modality, and the time of event was recorded as the date of imaging, when thromboembolic event was subjectively confirmed.

Cytogenetic studies were performed onsite, whose satisfactory performance was monitored by a national external quality assurance scheme. Bone marrow cells were cultured for 24 h then karyotype was analyzed using the standard G-banding technique. The karyotypes were constructed and chromosomal abnormalities were reported in accordance with the 2016 International System for Human Cytogenetic Nomenclature.

### Statistical analysis

The primary objective of this study was to investigate the incidence of thromboembolic events in homogeneous East Asian MPN patients per MPN subtype. MPN was divided into PV, ET, PMF and MPN-unclassifiable (MPN-U). The secondary objectives included the characteristics of thromboembolism, the effect of thromboembolism on survival, and indirect comparison with other ethnicities.

The incidence of a thromboembolic event was calculated at any time (within 12 months or at the time of MPN diagnosis, and during the follow-up). Categorical variables were summarized with the frequencies in number and rates in percentages. Continuous variables were represented with the median values and ranges. Differences were assessed using Mann–Whitney test for continuous variables and Pearson’s χ2 or Fisher’s exact test for categorical variables. The multiple imputation approach was applied to the missing values of BMI in 82 patients. Of multiple sets of data imputed, the set that had lowest standard error was chosen. Then obesity and the number of cardiovascular risk factors were recalculated upon the newly imputed data. Statistical analyses with the multiple regression model were reconstructed from the newly imputed data. While all patients had information on *JAK2V617F* mutations, 196 patients lacked *MPL* data and 192 patients lacked *CALR* data. Because more than one third of the patients lacked these data, *MPL* and *CALR* variables were not considered in this study. Multivariable Cox proportional-hazard models were constructed to find risk factors for thromboembolic events and correlation among covariates. False Discovery Rate approach with Benjamini–Hochberg procedures was used, and q-value < 0.05 was considered statistically significant. Since cardiovascular risk factors have been well defined in previous study^5^, instead of examining each of the cardiovascular risk factors as separate variables in the regression model, ‘two or more cardiovascular risks’ was considered to be an important risk factor. Since some variables are well-known risk factors with established threshold for the thrombotic events, they were dichotomized for multiple regression model. For example, age over 60 years old is a well-known risk factor for PV^2^. BMI was specifically defined as categorical variable because obesity criteria for Korean population was defined as BMI over 25. Leukocytosis is another widely studied risk factor. ECLAP study had shown the association between leukocytosis over 15 × 10^9^/L with the increase of venous thrombosis in PV patients^18^. Thus, these variables were converted into categorical variables. Other continuous variables such as hemoglobin and platelet counts were handled as quantitative values in the multiple regression model.

Overall survival (OS) was defined as the time from MPN diagnosis to death of any cause. The OS curves were estimated using the Kaplan–Meier method. If patients survived without death, the survival was censored at the latest date of follow-up when no death was confirmed. *p*-values of < 0.05 were considered statistically significant for Kaplan–Meier curves. These data were analyzed using the Statistical Package for the Social Sciences software (IBM SPSS Statistics, Version 22.0, New York, NY, USA).

Cumulative incidence of thromboembolic events of the MPN disease and subgroups was estimated taking into account the first event and considering death as a competitor and compared by Gray’s test. For this part of the analyses, the statistical software R (www.r-project.org) was used.

## Results

### Patient characteristics and treatment

The baseline characteristics are shown in Table 1. Among 256 MPN patients, the most common diagnosis was ET (42.2%, 108/256), followed by PMF (27.7%, 71/256) and PV (24.6%, 63/256). The median age at diagnosis for the entire cohort was 62 years (range, 19–88) and *JAK2V617F* mutation was positive in 73.0% (187/256). *MPL* mutation was tested in 60 patients and was positive in 2 ET patients and 1 MPN-unclassifiable (MPN-U) patient. *CALR* mutation was available in 64 patients, and was found positive in 5 ET, 10 PMF, and 1 MPN-U patient.

**Table 1:** Patient characteristics.

	Total	PV	ET	PMF	MPN-U
N (%)	256	63 (24.6%)	108 (42.2%)	71 (27.7%)	14 (5.5%)
Sex (female, %)	129 (50.4%)	35 (55.6%)	59 (54.6%)	30 (42.3%)	5 (35.7%)
Age at dx (median, range)	62 (19–88)	63 (41–86)	60 (19–88)	61 (25–86)	72 (36–84)
BMI (median, range)	23.7 (14.2–45.1)	24.2 (18.9–45.1)	23.9 (15.6–39.0)	22.7 (14.2–29.4)	23.2 (18.8–27.3)
Chromosomal abnormalities	29 (11.3%)	6 (11.5%)	7 (7.1%)	16 (22.5%)	0 (0.0%)
*JAK2V617F* (%)	187 (73.0%)	60 (95.2%)	69 (63.9%)	45 (63.4%)	13 (92.9%)
WBC × 10^9^/L (median, range)	10.2 (1.8–85.4)	13.3 (6.3–34.7)	9.1 (3.9–25.9)	10.3 (1.8–38.3)	14.1 (6.9–85.4)
Hemoglobin g/dL (range)	14.5 (5.7–25.9)	18.6 (13.2–25.9)	13.9 (9.1–17.7)	12.2 (5.7–17.2)	16.2 (9.5–21.2)
Hematocrit (range)	44.3 (17.4–80.0)	57.9 (41.4–74.6)	42.2 (27.9–54.4)	39.3 (17.4–80.0)	48.3 (31.0–64.7)
Platelet × 10^9^/L (range)	678 (15–2424)	440 (98–1322)	817 (328–2240)	577 (15–2424)	688 (345–1149)
Hepatomegaly (%)	11 (4.3%)	2 (3.2%)	1 (0.9%)	8 (11.3%)	0 (0.0%)
Splenomegaly (%)	74 (28.9%)	19 (30.2%)	12 (11.1%)	39 (54.9%)	4 (28.6%)
CVD risk (%)*	82 (32.0%)	18 (28.6%)	40 (37.0%)	17 (23.9%)	7 (50.0%)

* Patients who had two or more cardiovascular disease risk factors; CVD risks are defined as obesity (BMI > 25), smoking, hypertension, diabetes and dyslipidemia.

Dx, diagnosis; BMI, body mass index; WBC, white blood cell; CVD, cardiovascular disease.

Antithrombotic therapy was done in 84% (215/256) of MPN patients. Aspirin was most often used but various antithrombotic agents including clopidogrel, triflusal, prasugrel, dabigatran, and ticagrelor. In each subgroup, 94% (59/63) of PV, 85% (92/108) of ET, 56% of PMF (40/71), and 86% (12/14) of MPN-U were treated with antiplatelets. In PV patients, 79.4% (50/63) received phlebotomy. Hydroxyurea was prescribed to 83% (52/63), 77% (83/108), 47% (33/71), and 86% (12/14) of PV, ET, PMF, and MPN-U, respectively. Anagrelide was prescribed to 15.9% (10/63), 69.4% (75/108), 25.4% (18/71), and 35.7% (5/14) of PV, ET, PMF, and MPN-U patients, respectively. Ruxolitinib was prescribed to 41% (29/71) of the PMF patients.

At the time of diagnosis, 164 patients had one of the CVD risk factors (Table 1): 59/174 (33.9%) patients had BMI over 25, 33/256 (12.9%) patients were active smokers, 89/256 (34.8%) patients had hypertension, 32/256 (12.5%) patients had diabetes, 15/256 (5.9%) patients had underlying dyslipidemia.

### Incidence of thromboembolic events

**Figure 1:**
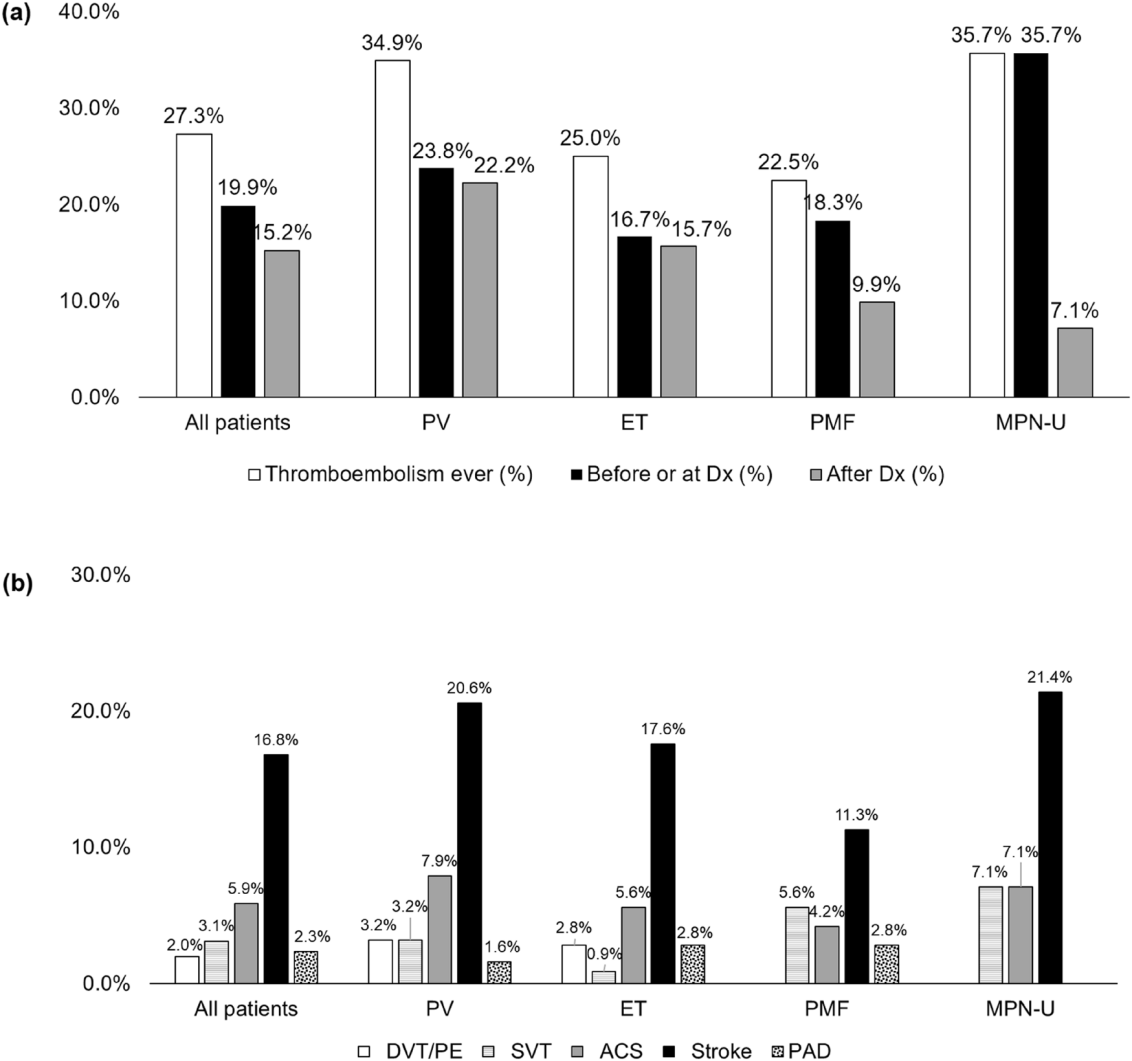
Overview of thromboembolic events in Korean myeloproliferative neoplasm (MPN) patients. (**a**) Per time point by MPN subtypes. (**b**) Site of thromboembolic events by MPN subtypes.

As shown in Fig. 1, 27.3% (70/256) experienced thromboembolic events. In subgroup analysis, MPN-U patient had the highest rate of thrombosis (35.7%, 5/14), followed by PV (34.9%, 22/63), ET (25%, n = 27/108) and PMF (23%, n = 16/71). For all MPN patients, the cumulative incidence of thromboembolic events was calculated as 6.3 (95% CI 3.3–9.3) at 1 year, 15.3 (95% CI 10.9–19.7) at 5 years, and 28.3 (95% CI 22.8–33.8) at 10 years. In subgroup analysis, the cumulative incidence rate of PV was 11.1 (95% CI 3.3–18.9) at 1 year, 20.3 (95% CI 10.4–30.2) at 5 years, 32.1 (95% CI 20.6–43.6) at 10 years. For ET, the cumulative incidence rate was 3.7 (95% CI 0.1–7.3) at 1 year, 15.2 (95% CI 8.4–22) at 5 years, 26.3 (95% CI 18–34.6) at 10 years. For PMF, the cumulative incidence rate was 5.7 (95% CI 0.3–11.1) at 1 year, 9.2 (95% CI 2.5–15.9) at 5 years.

### Site and characteristics of thromboembolic events

The sites and timing of thromboembolic events are shown in Fig. 1. Majority of the patients (72.8%, 51/70) experienced thromboembolic events within 12 months before or at the time of MPN diagnosis. Three patients experienced recurrent thrombosis, and all were *JAK2* positive ET patients. First patient had underlying protein C deficiency and suffered from alternating bleeding and thrombotic events. Second patient had concomitant chronic renal failure unrelated to ET, and despite continuous low dose aspirin treatment, the patient experienced multiple arterial thrombosis and stroke events 7 years after ET diagnosis. The last patient had not underlying disease but suffered from coronary artery disease and then peripheral artery disease despite the use of clopidogrel and cilostazol.

Stroke (16.8%, 43/256 was the most common cause of thrombosis, followed by ACS (5.9%, 15/256), and SVT (3.1%, 8/256). DVT or PE occurred in only PV and ET. SVT was particularly prevalent in the PMF (6.6%, 4/71) and the MPN-U (7.1%, 1/14) patients.

No patients experienced sinus vein thromboses or arm vein thrombosis. As for arterial events, 1 patient experienced tibial artery occlusion and 4 experienced splenic infarction.

### Risk factors of thromboembolic events

For all MPN patients, multivariable analysis identified leukocytosis (HR 2.67, 95% CI 1.36–5.24, *p* = 0.004), and history of thrombosis (HR 5.24, 95% CI 2.73–10.08, *p* < 0.001) as risk factors for thromboembolism as shown in Table 2.

**Table 2:** Risk factors for thromboembolic events.

Disease	Variable	HR (95% CI)	q-value
MPN	Age at dx	2.08 (1.01–4.26)	0.045
	Sex	1 (0.52–1.95)	0.993
	Leukocytosis*	2.67 (1.36–5.24)	0.004
	Hemoglobin	1.03 (0.93–1.13)	0.564
	CVD risk factor**	1.48 (0.76–2.87)	0.249
	History of thrombosis	5.24 (2.73–10.08)	< 0.001
PV	Age at dx	2.79 (0.52–15.04)	0.233
	Sex	3.49 (0.63–19.24)	0.151
	Hepatomegaly	19.9 (0.79–498.94)	0.069
	Splenomegaly	0.58 (0.13–2.5)	0.466
	Leukocytosis*	5.91 (0.74–47.08)	0.093
	Hemoglobin	1.97 (1.28–3.04)	0.002
	Platelet	1 (1–1.01)	0.341
	CVD risk factor**	0.37 (0.05–2.73)	0.331
	History of thrombosis	9.68 (2–46.88)	0.005
ET	Age at dx	1.76 (0.55–5.63)	0.342
	Sex	1.2 (0.44–3.29)	0.72
	Splenomegaly	3.13 (0.67–14.6)	0.15
	JAK2V617F	0.48 (0.17–1.4)	0.181
	Leukocytosis*	2.65 (0.45–15.62)	0.281
	Platelet	1 (1–1)	0.354
	CVD risk factor**	1.95 (0.61–6.2)	0.258
	History of thrombosis	5.16 (1.73–15.38)	0.003
PMF	Age at dx	3.82 (0.32–45.61)	0.29
	Sex	0.39 (0.03–4.85)	0.466
	Hepatomegaly	1.21 (0.04–32.51)	0.911
	Splenomegaly	1.29 (0.17–9.64)	0.805
	JAK2V617F	0.49 (0.03–8.3)	0.621
	Leukocytosis*	4.34 (0.54–34.9)	0.167
	Platelet	1 (1–1)	0.823
	CVD risk factor**	2.29 (0.34–15.63)	0.397
	History of thrombosis	13.85 (1.2–g159.5)	0.035

* Patients who had two or more cardiovascular disease risk factors; CVD risks were defined as obesity (BMI > 25), smoking, hypertension, diabetes and dyslipidemia.

** Leukocytosis was defined as WBC > 15 × 10^9^/L.

HR, Hazard ratio; CI, confidence interval; MPN, myeloproliferative neoplasms; PV, polycythemia vera; ET, essential thrombocythemia; PMF, primary myelofibrosis; dx, diagnosis; BMI, body mass index; WBC, white blood cell; CVD, cardiovascular disease.

**Figure 2:**
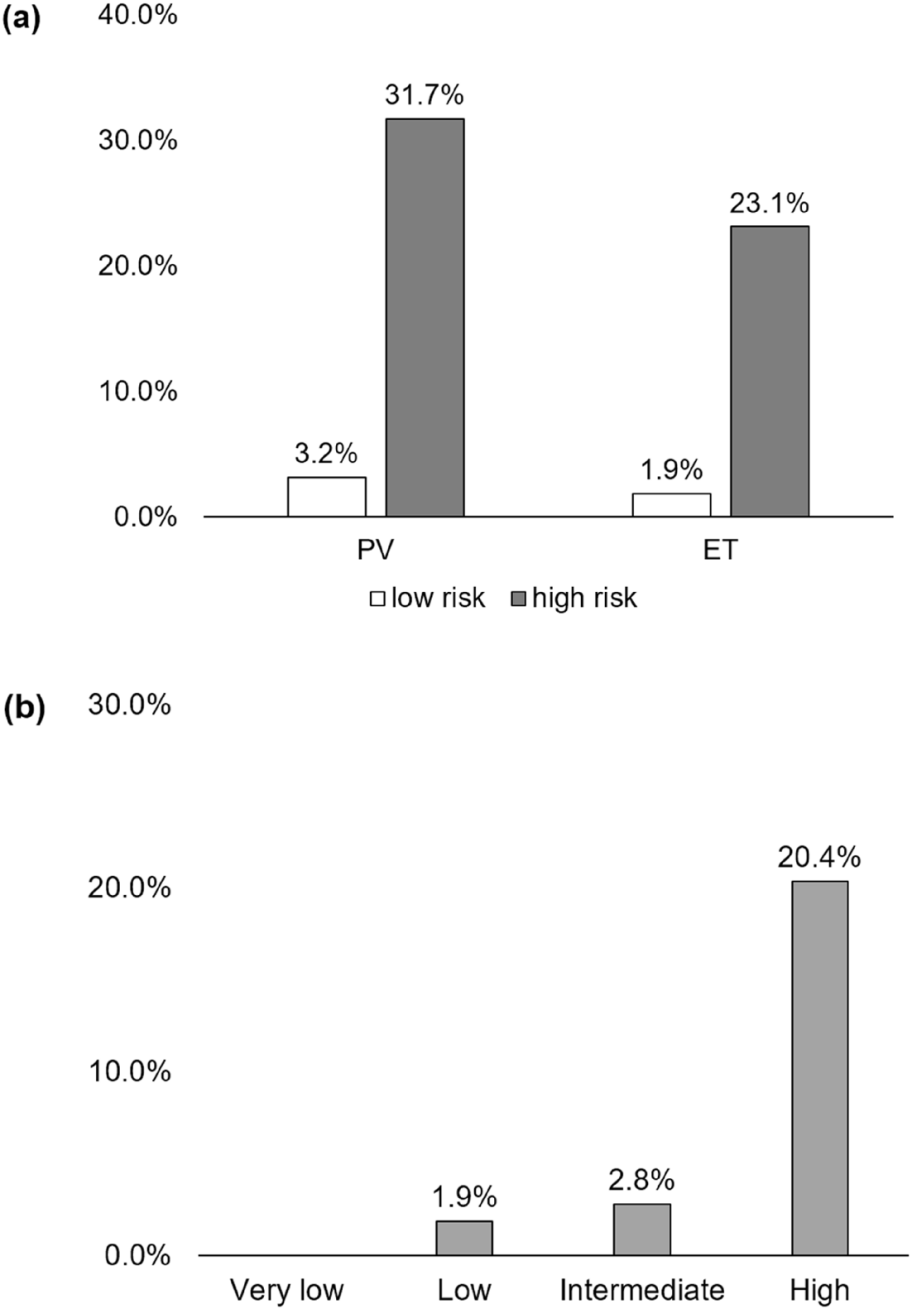
Thromboembolic events according to risk stratification. (**a**) Polycythemia vera and essential thrombocythemia (ET) patients according to conventional risk stratification according to age and history of thrombosis. (**b**) ET patients IPSET-thrombosis risk stratification.

The risk stratification of PV and ET is based on the age (older than 60 years) and history of previous thrombosis^19,20^. When PV patients were classified accordingly, thrombosis occurred in 31.7% of high-risk group, in contrast to 3.2% in low-risk group, as shown in Fig. 2a. In the subgroup analysis of PV patients, the history of thrombosis (HR 9.68, 95% CI 2.00–46.88, *p* = 0.005), and hemoglobin count (HR 1.97, 95% CI 1.28–3.04, *p* = 0.002) were recognized as risk factors for thrombosis in multivariable analyses, as shown in Table 2.

When ET were classified according to traditional risk stratification, thrombosis occurred in 23.1% of high-risk group, in comparison to 1.9% in low-risk group as shown in Fig. 2a. When the ET patients were analyzed by International Prognostic Score of Thrombosis for Essential Thrombocythemia (IPSET)^19^, thrombosis rate increased from 1.9% in low risk to 20.4% in high risk as shown in Fig. 2b. It is also notable that thrombosis prior to ET diagnosis only occurred in the high-risk groups. The multivariable Cox proportional model for ET patients showed that the history of thrombosis was associated with increased thrombosis events, as in Table 2.

For PMF patients, history of thrombosis was associated with increased thrombosis events in multivariable analysis, as shown in Table 2.

### Survival

**Figure 3:**
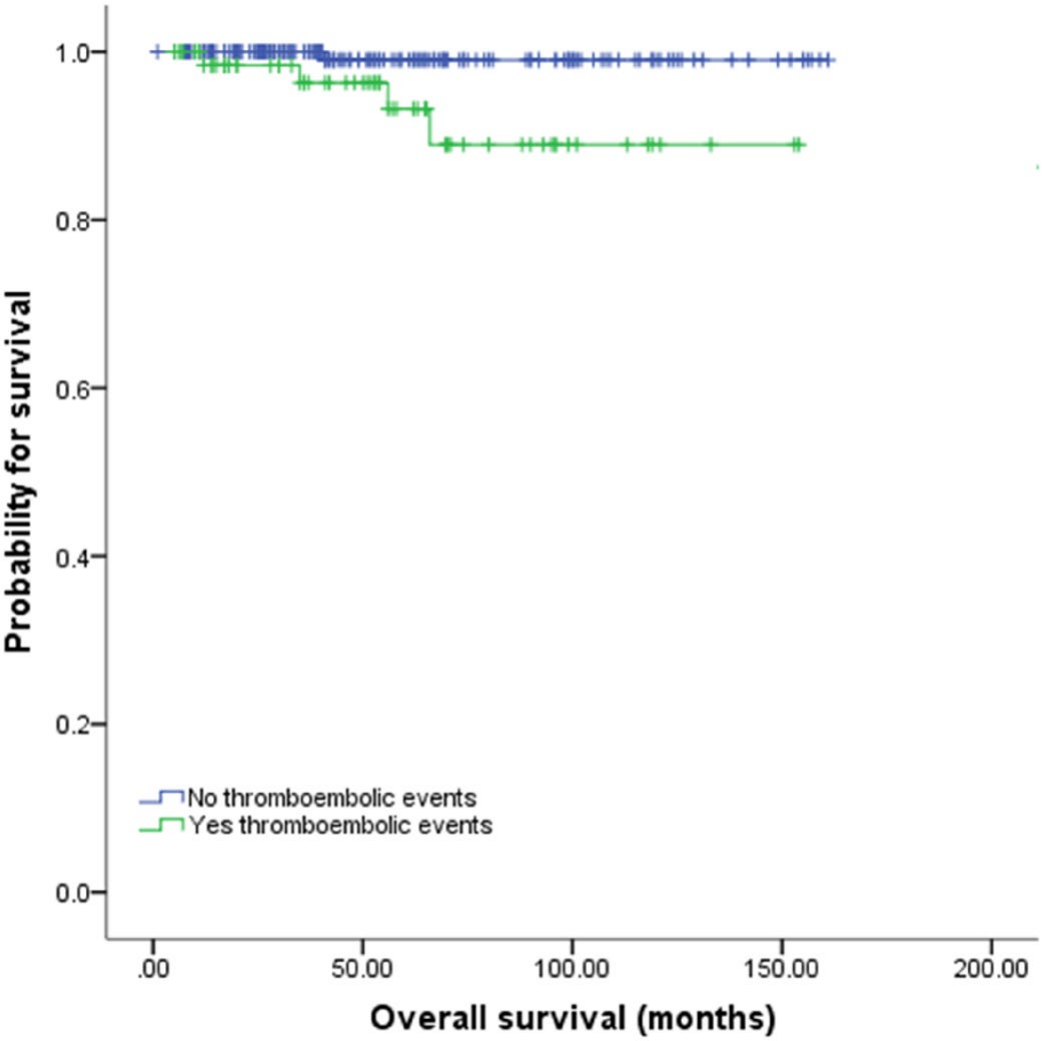
Overall survival according to thromboembolic events.

The follow-up period for each MPN subtypes were 49 months, 64 months, 35 months, and 33 months for PV, ET, PMF, and MPN-U, respectively. The number of the patients followed up at 1 year, 5 years and 10 years were 136, 77, and 21, respectively. During the median follow-up of 46 months (range 12–161), 5 patients experienced secondary transformation: 2 to acute myeloid leukemia (1 ET, time to progression 43 months; 1 PMF, time to progression 7 months) and 3 to secondary myelofibrosis (1 PV, time to progression 127 months; 2 ET, median time to progression 63 months). The estimated overall survival for the entire cohort was 96.2%. While PV patients were associated with best survival and PMF patients with worst survival, the difference did not reach statistical difference. As shown in Fig. 3, patients with thromboembolic events had shorter overall survival compared to those who did not (*p* = 0.009).

## Discussion

The importance of our study lies in that (1) we present rare data on the incidence and characteristics of East Asian patients, which seem to be similar to Western data albeit the traditional belief that Asians are less prone to thromboembolism; (2) we report risk factors for thromboembolism; and that (3) management of thromboembolism can lead not only to better quality of life but also better survival.

As shown in Table 3, our study provides evidence that Korean patients had similar frequency of thrombosis compared to that of Western patients, ranging 16 ~ 41%^19,23,24,28,31–33^. More specifically, recent meta-analysis of 29 cohort studies including populations of Europe, North America, Asia, and Australia reported that the pooled prevalence of arterial or venous thrombosis among MPN patients at diagnosis was 20% (95% CI, 16.6–23.8%)^12^. In our study, 27.3% of the patients experienced overall thrombosis, within close range of other ethnic populations. Also in subgroup analysis, despite the diverging rates of the thromboembolic events among multiple ethnic groups, our data provided the evidence that the Asians were not of lower risk of vascular complications. It is also notable that over one third of MPN-U patients experienced thromboembolism among all subgroups, albeit the small number of populations and the scarcity of the preceding data. When compared by the site of the thrombosis, stroke and ACS were the most prevalent but thromboses in uncommon sites like SVT were also reported, shown in Table 4. Markedly high prevalence of stroke and its decrease during the follow-up period indicated the role of the treatment with antiplatelet agents in reducing vascular complications in Asian MPN patients. Considerable number of patients who were diagnosed with MPNs within a month of thrombotic events also suggested the need for the early diagnosis and treatment to prevent vascular complications, as observed in the ECLAP study and the German MPN registry^13,18^.

**Table 3:** Comparison of thrombosis before diagnosis of MPN.

	Ethnicity	N	Total (%)	PV (%)	ET (%)	PMF (%)	MPN-U (%)
Current study	Korean	256	19.9	23.8	16.7	18.3	35.7
Enblom^22^	European	612	24.6	41.0	44.0	15.0	–
Veroli^26^	Italian	1087	16.0	16.8	16.4	13.6	–
Kaifie^21^	German	438	33.6	38.9	25.0	31.2	27.8
Soyer^29^	Turkish	708	15.9	20.7	15.1	9.5	–
Abdulkarim^30^	Swedish	2350	34.9	37.0	35.0	–	–
Szuber^17^	U/A	3023	20.0	22.0	35.0	42.0	–
Seguro^31^	Brazilian	334	41.0	27.0	46.0	21.0	–

**Table 4:** Comparison of thrombosis site.

	Ethnicity	N	ACS (%)	Stroke (%)	DVT/PE (%)	SVT (%)	PAD (%)
Current study	Korean	256	5.9	16.8	2.0	3.1	2.3
Enblom^22^	European	612	7.5	10.6	3.1	1.1	1.1
Kaifie^21^	German	438	9.4	6.4	10.5	5.0	3.3
Abdulkarim^30^	Swedish	2350	6.1	10.9	4.3	1.4	3.3
Seguro^31^	Brazilian	334	12.0	17.1	9.6	10.5	3.6

MPN, myeloproliferative neoplasms; PV, polycythemia vera; ET, essential thrombocythemia; PMF, primary myelofibrosis; ACS, acute coronary disease; DVT, deep vein thrombosis; PE, pulmonary embolism; SVT, splanchnic vein thrombosis; PAD, peripheral artery disease.

Risk factors for thromboembolism varied across subtypes of MPN, emphasizing the importance of individualized treatment depending on the subtype, presentation and comorbidities. PV was associated with the highest incidence of thromboembolism among MPN subgroups, comparable to previous observations that reported 26–39%^19,22,23^. In addition, that hemoglobin concentration was markedly associated with thrombosis events implied that effective control of hyperviscosity with phlebotomy is required to control the disease and reduce the vascular complications^23^. In ET, the severity of IPSET score was predictive of thromboembolic events. Therefore, ET patients should be treated more comprehensively including management of cardiovascular comorbidities to prevent thromboembolism in ET patients. In PMF, age over 60 years, *JAK2V617F* mutational status, and previous thrombosis had been identified as risk factors of thrombosis^26,27^. In our study, however, history of thrombotic event was the only predictive variable on multivariable analysis that included age, sex, organomegaly, *JAK2V617F* mutation status, blood counts, and cardiovascular risk factors as covariates (*p* = 0.035). The shorter overall survival of the patients with thromboembolic events underscored the importance of recognizing high risk patients and implementing personalized intervention.

One of the most obvious pitfall of the study is its retrospective nature. Also, there is a possibility of referral bias, since all participating hospitals are academic centers. However, considering the longevity of MPN patients, prospective data is very difficult to accrue. The small number of MPN-U patients may have overestimated the prevalence of thromboembolism which was comparable to that of the PV, and the rate of splanchnic vein thrombosis (7.1%, 1/14). However, preliminary data showed that MPN-U patients with normal blood count may present with rare unexplained thrombosis, especially in splanchnic vein^28,29^. Lastly, to compensate for the changes in the MPN diagnostic criteria after 2015, we selected patients with legitimate bone marrow examinations results and initial laboratory findings. Thus, it is our assumption that patients were little affected by the changes in the diagnostic criteria. Previous studies have compared the difference in thrombosis events of patients who were diagnosed with 2008 versus 2016 criteria^34,35^. The major updates in the 2016 WHO classification for Philadelphia-negative MPN aimed at distinguishing between masked PV and *JAK2*-mutated ET, and between prefibrotic and overtly fibrotic PMF. In PV, the reduced hemoglobin level threshold unveiled that up to 72% of the patients newly diagnosed with prodromal/masked PV had history of thromboembolism. On the other hand, pre-PMF is known to have comparable cumulative incidence of major thrombosis to that of ET. Unfortunately, since there are only 28 PV, 66 ET, 23 PMF, and 6 MPN-U patients were diagnosed before 2015, subgroup comparison analysis was not carried out. Further studies with more patient data on the masked PV and pre-PMF would strengthen our knowledge in understanding the vascular complications of MPN patients. All in all, a descriptive epidemiology study can assist public health planning, policy making, fair allocation of limited healthcare resources, and understanding of disease secular trends. In this regard, regardless of study limitations, it is our belief that our study holds its values.

## Conclusion

Our study provides better understanding of the epidemiology, characteristics and risk factors of thromboembolic events in East Asian patients with MPN, who have been underrepresented in previous studies. Meticulous risk factor evaluation is crucial for prevention of thrombosis and better survival.

## Data Availability

The datasets generated during and/or analyzed during the current study are available from the corresponding author on reasonable request.
